# The remarkably frequent use of EQ-5D in non-economic research

**DOI:** 10.1007/s10198-021-01411-z

**Published:** 2021-11-30

**Authors:** Aimin Wang, Kim Rand, Zhihao Yang, Richard Brooks, Jan Busschbach

**Affiliations:** 1 School of Medical Management, Shandong First Medical University & Shandong Academy of Medical Sciences, Tai’an, China; 2 Section Medical Psychology and Psychotherapy, Erasmus Medical Center, Rotterdam, The Netherlands; 3 Health Services Research Center, Akershus University Hospital, Lørenskog, Norway; 4 Math in Health B.V., Rotterdam, The Netherlands; 5 Health Services Management Department, Guizhou Medical University, Gui’an, China; 6 EuroQol Group Research Foundation, Rotterdam, The Netherlands

**Keywords:** EQ-5D, Non-economic use, Economic use, HRQoL

## Abstract

**Introduction:**

EQ-5D is an instrument which has been utilized for a variety of purposes, including in health-economic appraisals as an input into quality-adjusted life year (QALY) calculations. Indeed, it is the most-widely applied instrument for health-economic appraisal worldwide, and is recommended for use in QALY calculations by many national Health Technology Assessment (HTA) agencies. There is also a growing body of evidence for its usefulness in a variety of settings other than economic appraisals, but such use has not been well-documented. This study addresses this issue and documents how EQ-5D has been applied in both the non-economic and economic contexts.

**Methods:**

The PubMed database was searched using the terms ‘EQ-5D’, ‘EQ-5D AND cost’, and ‘EQ-5D AND cost AND QALY’ from 1 January 1980 to 31 December 2019. We concentrated on 2019 publications for more detailed analyses. All the data collected for 2019 were downloaded and collected in EndNote. For 2019 only, we classified economic and non-economic use based on the inclusion of ‘cost’. We also checked by manual inspection whether the search terms were suitable in correctly identifying economic and non-economic use. Variants of the non-economic use of EQ-5D were classified as follows: (a) as a quality of life outcome measure; (b) as a tool for methodological research; (c) methodological issues of EQ-5D itself; (d) comparisons with other quality of life questionnaires; (e) mapping studies; (f) value sets; (g) alongside costs but no QALY calculated; and (h) other.

**Results:**

The first publication found was from 1990. Up to and including 2019, 10,817 publications were identified, of which more than two in three did not contain any reference to costs or QALYs. In 2019, a total of 1409 manuscripts were identified, of which 239 were specifically for EQ-5D-5L.  Four hundred and seven (28.9%) included some form of ‘costs’ and 157 (11.1%) both ‘costs’ AND ‘QALYs’ terms. For EQ-5D-5L, the corresponding numbers were 104 (43.5%) and 29 (12.1%), respectively. After manually checking all the 1409 papers, three were duplicated records, which were omitted. In the remaining 1406 papers, only 40 (2.8%) contained the term ‘cost’, but not ‘cost per QALY’, and only 117 (8.3%) were identifiable as economic evaluations using the term ‘cost per QALY’. Most non-economic use of EQ-5D was as a quality-of-life outcome measure (72.8%). Other applications were: as a tool for methodological research (6.7%); comparison studies (3.7%); EQ-5D methodological issues (3.5%); containing costs but not QALYs (2.8%); mapping (1.3%); value sets (0.4%); and other papers (0.4%).

**Conclusions:**

The majority of the studies retrieved, covering a wide variety of research areas, reported upon the non-economic use of EQ-5D. Despite being the most-used instrument worldwide for QALY calculations, economic appraisal accounted for only a small, but important, part of published use.

## Introduction

The EQ-5D instrument is a health-related quality-of-life (HRQoL) questionnaire which has been extensively used for a variety of purposes around the world [1], including a wide range of clinical studies, randomized controlled trials, and population health surveys. It has also been the most widely-used HRQoL instrument in health economic evaluations [2].

EQ-5D facilitates economic evaluation in the form of cost–utility analysis, as it is a generic, instrument capable of deriving values (sometimes termed ‘utilities’) for health states which can then be used to estimate quality-adjusted life years (QALYs). EQ-5D has been widely adopted, and plays an important role in assessing the comparability, transparency, and consistency of economic evaluations in informing resource allocation in healthcare [3]. In a recent review of official national pharmacoeconomic guidelines, it was concluded that EQ-5D was, by far, the most frequently recommended instrument: in 85% of the guidelines, EQ-5D was either the preferred instrument to measure HRQoL or as an example instrument utilized in cost–utility analysis [4].

While this is evidence of the successful uptake of EQ-5D in the context of economic evaluation, there is a lack of knowledge regarding its use for a wide variety of other purposes. This study aimed to perform the first detailed estimation of the uses of EQ-5D for purposes other than economic evaluation. This paper: (i) calculates the relative use of EQ-5D in non-economic and health economic studies and (ii) describes and applies a classification system for the analysis of EQ-5D-related studies.

## Methods

To analyze the use of EQ-5D in both non-economic and economic publications over time, we performed a longitudinal exploration of publications mentioning EQ-5D. The search was restricted to the PubMed database. Included were both papers and letters to the editors that reported on original research. Commentaries on papers were excluded. Articles in languages other than English were excluded if the accompanying English abstract did not provide sufficient information for classification. The search terms ‘EQ-5D’, ‘cost’, and ‘QALY’ were used to obtain a picture of the number of publications that mentioned EQ-5D in relation to economic analyses. Articles published in the final year of the time frame employed were studied in detail: we manually examined the title and abstract of all publications identified in 2019, to investigate the non-economic use of EQ-5D. This was undertaken to determine the relative proportions of published papers that used EQ-5D for economic and non-economic purposes. In this way, we could validate the estimates obtained using search terms alone. In addition, we constructed a system to classify and tabulate the non-economic uses of EQ-5D.

To determine the pool of relevant papers, we searched for EQ-5D and related terms: ‘EQ-5D’, ‘EQ5D’ and ‘EuroQol’. To allow for compound words, we added a ‘wildcard’ after the search terms: ‘EQ5D*’. This made the first query: (EQ-5D* OR EQ5D* OR EQ 5D* OR EuroQol*). A second query added the search terms (cost OR cost*), to find all articles that used EQ-5D with respect to costs. This was used as a broad indicator of an ‘economic context’. Hence, the query became: (EQ-5D* OR EQ5D* OR EQ 5D* OR EuroQol*) AND (cost OR cost*). For the third query, we narrowed the economic context to QALYs: (EQ-5D* OR EQ5D* OR EQ 5D* OR EuroQol*) AND (cost OR cost*) AND (QALY OR QALY*). The search words above applied to title, abstract and contents of the papers.

These queries produced hits from 1990 onwards, as in that year the first article describing what was then called ‘the EuroQol instrument’ was published [5]. The term ‘EQ-5D’ was introduced in 1995**.** After collecting yearly statistics until to the end of 2019, based on these three searches, data collection was terminated on May 15, 2020. This was necessary, as changes are continuously made to the PubMed database, through the addition of new journals, old archives being added, reclassification of publication years, etc. The titles and abstracts of the 2019 publications were imported into EndNote for the manual analysis and this information is available on request.

Most papers identified related to the original version of EQ-5D. In 2007, the first two papers concerning the EQ-5D-5L version were published [6, 7], and the original version was often referred to as EQ-5D-3L. A separate analysis was undertaken for this new 5L version.

All the titles and abstracts for the year 2019 were checked manually. If an article included both the search terms ‘QALY’ and ‘cost’, we checked whether the article did indeed report a cost per QALY ratio, to observe whether EQ-5D was used in a cost-effectiveness context, and not just as a way of reporting a QALY independently of costs. The paper searched was excluded if it did not refer to ‘QALY’ and ‘cost’. When checking the 2019 papers, we also categorized them in terms of the nature of the application.

## Results

**Fig. 1:**
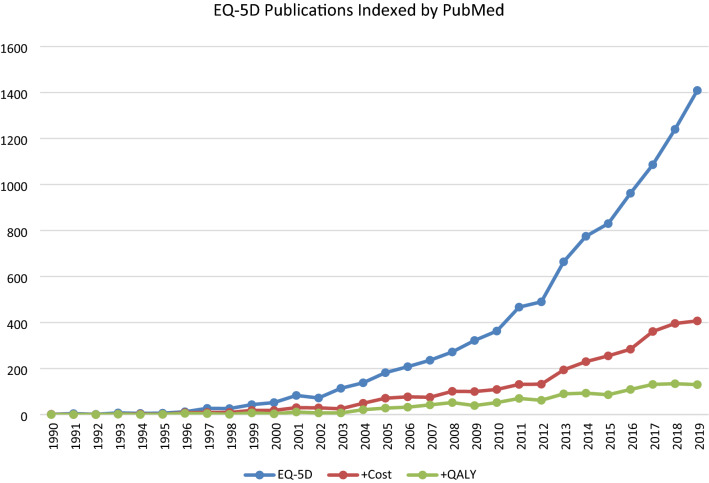
EQ-5D publications indexed by PubMed. Number of articles over the years referring to both 3- and 5-level versions of ‘EQ-5D* OR EQ5D* OR EQ 5D* OR EuroQol*’ (blue); ‘(EQ-5D* OR EQ5D* OR EQ 5D* OR EuroQol*) AND (cost OR cost*)’ (red); ‘(EQ-5D* OR EQ5D* OR EQ 5D* OR EuroQol*) AND (cost OR cost*) AND (QALY OR QALY*)’ (green). The graph is not ‘stacked’. Therefore, the articles that contained ‘(EQ-5D* OR EQ5D* OR EQ 5D* OR EuroQol*) AND (cost OR cost*) AND (QALY OR QALY*)’ were also present in ‘(EQ-5D* OR EQ5D* OR EQ 5D* OR EuroQol*) AND (cost OR cost*)’. First article appeared in 1990

From 1990 to 2019, 10,817 papers were found using the EQ-5D search terms: (EQ-5D* OR EQ5D* OR EQ 5D* OR EuroQol*). Figure 1 shows that over the years, the average ratio of papers involving EQ-5D with costs was exactly one in three (0.33). Articles that made a link to QALYs were on average one in eight (0.124) of the number of EQ-5D papers. These ratios per year remained remarkably constant over time.

**Fig. 2:**
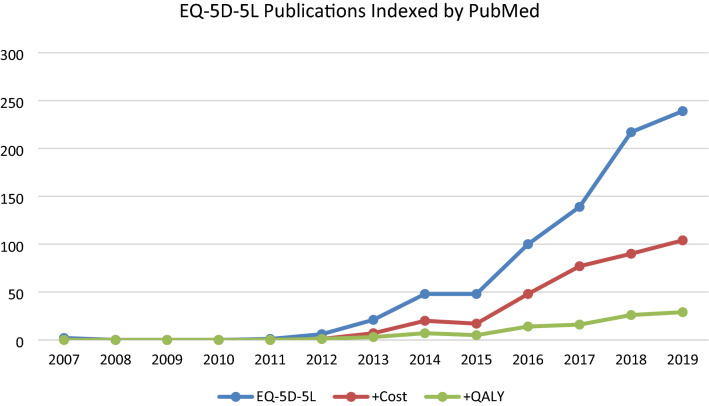
EQ-5D-5L publications indexed by PubMed. Number of articles over the years referring to ONLY ‘EQ-5D-5L* OR EQ5D-5L* OR EQ 5D-5L*’ (blue); ‘(EQ-5D-5L* OR EQ5D-5L* OR EQ 5D-5L*) AND (cost OR cost*)’ (red); ‘(EQ-5D-5L* OR EQ5D-5L* OR EQ 5D-5L*) AND (cost OR cost*) AND (QALY OR QALY*)’ (green). First article appeared in 2007

Similar observations can be seen in Fig. 2, which presents the results for the EQ-5D-5L version only. Again, over the years the average ratio of the papers involving EQ-5D-5L related to costs was approximately one in three (0.32), and the articles that made a link to QALYs were on average one in nine (0.11) of the EQ-5D-5L papers.

**Fig. 3:**
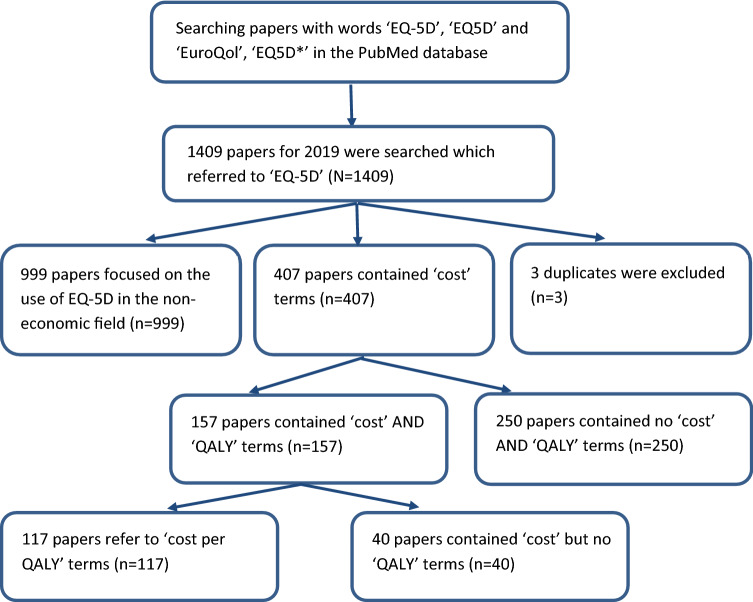
Flowchart of study selection

For 2019, there were 1409 papers, of which 407 (28.9%) contained ‘cost’ terms and 157 (11.1%) contained ‘cost’ AND ‘QALY’ terms (see Fig. 3). For EQ-5D-5L the corresponding numbers were 239 (total), 104 containing ‘cost’ (43.5%) terms and 29 containing ‘cost’ AND ‘QALY’ terms (12.1%). After checking the 1409 papers for 2019, six papers were not in English language, but the accompanying abstract provided sufficient information for categorization. Three duplicates were excluded. The search terms covering ‘cost’ and ‘QALY’ overestimated economic applications of EQ-5D in the remaining 1406 papers for the year 2019. As reflected in Table 1 and Fig. 3, only 40 papers (2.8%) actually described cost(s) but not QALYs, and 117 (8.3%) described economic evaluations using ‘cost per QALY’ calculations, for a joint total of 157 papers. This indicates that to count in the sample the papers including terms such as ‘cost’ or ‘QALY’ overestimates the proportion of EQ-5D papers relating to health economic evaluations, and in 2019 only 1 out of 12 papers used EQ-5D to calculate cost per QALY.

**Table 1:** Application, methodologic and development categories of EQ-5D

Categories	Numbers	Proportions (%)
Classical HRQoL outcome measure	1024	72.8
Tool for methodological research	94	6.7
Cost per QALY	117	8.3
Comparison	52	3.7
Methodological issues for EQ-5D itself	49	3.5
Alongside cost but QALY not calculated	40	2.8
Mapping	18	1.3
Value set	6	0.4
Other	6	0.4

The majority of the papers identified from 2019, 1289 (91.7%), focused on the use of EQ-5D in non-economic areas. When classifying the non-economic applications, it was difficult to construct mutually exclusive categories. For example, EQ-5D was often used in conjunction with other questionnaires, which could be either for comparison or for validation purposes. We arrived at eight categories that were considered the most informative.

The eight categories were:

- EQ-5D used as a HRQoL outcome measure in a clinical study. This included its use as a quality of life outcome in trials (randomized and non-randomized), burden of illness studies, and other epidemiological cohort studies such as patient reported outcome (PRO) or Routine Outcome Monitoring studies..
- Methodological research: EQ-5D presented as simply a tool, while something else was the real matter of interest, for example to validate another questionnaire.
- Methodological issues for EQ-5D itself: characteristics of EQ-5D investigated as the main objective of the research, with the intention of improving the instrument.
- EQ-5D in comparison with other quality of life instruments: tested against other instruments in patient cohorts to test comparative psychometric properties, such as validity and reliability.
- Mapping: EQ-5D utilities/values mapped onto a questionnaire not designed to calculate these.
- EQ-5D value sets.
- No cost per QALY ratio calculated: only cost and/or QALY estimated.
- Other (e.g., mislabeled).

The classification results are shown in Table 1 together with the economic application category ‘Cost per QALY’. References to examples of papers are given within each category.

The most common use of EQ-5D (72.8%) was as an HRQoL outcome measure in trials (randomized and non-randomized), cohorts, burden of illness, and other epidemiological studies.

(i) Clinical trial: EQ-5D was employed as a primary or secondary outcome in an investigation, where two groups of patients were compared, and one of the groups received an experimental treatment. This trial could be randomized [8–10], or not randomized [11, 12], where the control group received care-as-usual.
(ii) Burden of illness study: EQ-5D was used to express the severity of the disease and/or the relationship between the severity of the disease and the different medical and background variables that were examined [13, 14].
(iii) Cohort study: a group or different groups of patients were followed through time and their trajectories compared or related to an event that could change their quality of life, such as a transplantation or a traumatic occurrence [15, 16]. EQ-5D has been increasingly used as a routine outcome measure in clinical practice [17–19]. In these cohort studies, EQ-5D was usually administered at different time points, for example, at each treatment cycle during chemotherapy. The times of repeated measurement and the exact time points used for EQ-5D application depended on the study objective and study protocol. A common characteristic was that the trajectories of the subsequent HRQoL measures recorded could provide input for discussions between physicians and patients. Cohort data was also used to compare hospital wards employing different medical strategies. Typically, this type of data could then be used for benchmarking.

As a tool for methodological research EQ-5D was mainly used to estimate the validity of another HRQoL questionnaire [20, 21].

In cost per QALY studies, EQ-5D was indeed used to elicit utilities in the estimation of QALYs [22–24].

In comparison studies, the acceptability, validity, responsiveness, and other psychometric properties of EQ-5D and other instruments [25, 26], notably the Short-Form 6-Dimension (SF-6D) [27, 28], were compared.

With respect to those studies which investigated methodological issues concerning EQ-5D itself, these mainly explored the direct valuation of fewer health states in a value set study [29, 30], and the validity of EQ-5D in patients with specific diseases [31–33]. Notably, in 2019, investigations of possible ‘bolt-on’ dimensions for EQ-5D were prominent [34–36].

When EQ-5D was employed alongside cost estimates but QALYs were not calculated, it was typically used to calculate the burden in HRQoL [37–39], along with direct medical costs and indirect costs. In some studies, cost per QALY was not estimated, but the QALY calculations were presented anyway [40, 41].

Finally, a small number of studies mapped EQ-5D onto other instruments, and a few other papers derived value sets for EQ-5D [42, 43]. In this classification system, three categories, i.e., ‘Classical HRQoL outcome measure’, ‘Cost per QALY’ and ‘Alongside cost but QALY not calculated’ can be seen as application of EQ-5D and the rests of the categories are more related to the methodological aspect. This binary classification system also gives us insight about how EQ-5D was used.

## Discussion

### Summary

This is the first literature review of EQ-5D placed in the context of the instrument’s use in economic appraisals of health and medical activities compared with its application in a wide variety of non-economic contexts.

The most common use of EQ-5D was shown to be as a HRQoL outcome measure in trials (randomized and non-randomized), cohort studies, burden of illness studies, and other epidemiological studies.

### Interpretation

If a restrictive definition, namely, cost per QALY analysis, is used to characterize economic evaluation in health, only a very small proportion of EQ-5D papers fell into the economic evaluation category: in the year 2019 only 8.3% of the papers using EQ-5D were reports of cost per QALY analyses. Applying a less restrictive definition to papers deemed to have an economic context, this ratio increased somewhat. For example, there were articles that reported the ‘utility’ value of health states that could be used in future health economic modelling [3]. In addition, papers were retrieved concerning: (i) EQ-5D value sets, (ii) methodological aspects of EQ-5D, and (iii) the use of EQ-5D in mapping exercises that assisted other instruments to generate values suitable for QALY analysis. Moreover, there were papers that reported on costs and QALYs but did not strictly implement the cost per QALY approach. Hence EQ-5D was used more often in a health economics context than the strict definition of cost per QALY would imply, and many other papers played a role in providing the infrastructure for subsequent health economic appraisal. Nevertheless, a mere one third of the papers identified used the word ‘cost’ in the title, abstract or key words. From this alone it can be concluded that in approximately two out of three papers that mentioned EQ-5D, neither costs, nor more comprehensive economic appraisal, played prominent roles.

The EuroQol Group website (http://www.euroqol.org) states that EQ-5D can be used in clinical trials, population health surveys, routine outcome measurement, and many other types of studies, where a generic measure of health status can be usefully applied. Indeed, such a broad base of applications has been displayed in our research.

This widespread use and application of EQ-5D can be attributed to a number of factors [1].

(i) The development of a short and simple generic health status instrument which aimed to minimise the burden of both the measurement and valuation of health status led first to its adoption by medical personnel, and then its application in economic evaluation in health care, drug appraisals, and HTA.
(ii) Although for some years the EuroQol Group maintained that its instrument was to be used ‘alongside’ other instruments in evaluating medical programmes, and indeed this has often been the case, as shown above, EQ-5D increasingly came to be employed as a stand-alone instrument.
(iii) The business model employed by the Group generated revenue from commercial users with which to fuel further research, while allowing free use of the instrument by academics.
(iv) The ‘open access’ policy, including for Group publications, pursued from the outset.
(v) EQ-5D is backed by 30 years of data, evidence, publications, and researcher and user experience. This enables new data to be compared to EQ-5D population norms, EQ-5D evidence from specific disease groups, and so on.

Moreover, EQ-5D has demonstrated satisfactory psychometric properties in the assessment of patients, has high utility for demonstrating changes in disease activity and disability [33], is available in many languages, and has been validated in many countries.

It is worth recalling that the first clinical application of the EuroQol instrument was in 1993 [44]. As the number rose, the Group observed that pages 2 and 3 of its questionnaire, containing the descriptive system and EQ VAS, were being applied separately from the valuation section of the questionnaire, which aimed to provide valuations of health states for use in economic appraisal including the derivation of QALYs. The long-term increase in the use of EQ-5D as a HRQoL instrument is amply demonstrated in our study: almost 11,000 papers over a 30-year period. This is evidently a considerable under-estimate of its usage. It is predominantly, e.g., composed of English-language papers.

This paper has demonstrated a broad range of categories of EQ-5D usage, including a significant proportion of papers which do focus on economic aspects of health and medicine, in line with one of the original aims of the Group to develop a HRQoL instrument that could be used in valuing health and health interventions for the purposes of economic appraisal. We have also shown that applications of EQ-5D range well beyond the economic, and display an impressive richness and diversity, as exemplified by the category classification. It is especially evident that the instrument is a popular choice in the measurement of HRQoL, as emphasized in the proportion of papers retrieved in our search that used EQ-5D for this purpose.

### Limitations

Our research also has some limitations.

(i) The search was limited in that it was restricted to papers in data base, and the in-depth study of non-economics applications was limited to the year 2019. Hence some studies may have been missed. Specifically, (1) limiting the search to PubMed could lead to systematic omissions if some journals or some non-medical studies were not indexed on the PubMed portal; (2) some unpublished economic evaluation studies could be missed; (3) earlier studies not indexed in PubMed could be omitted. With respect to point (1), undertaking a systematic review following PRISMA guidelines would be expected to offer a more comprehensive picture of EQ-5D uses/applications.
(ii) Although all practicable efforts were made to ensure that the classification of the 2019 papers was valid, it is still possible that some papers were misclassified.
(iii) The categories adopted were assumed to be mutually exclusive, but this may not have fully matched reality: many papers could be classified as falling into two or more of the categories used here. For example, we noticed many protocol papers (*n* = 87) and we classified these protocol papers based on their intended use of EQ-5D. Sometimes there were multiple intentions for the use of EQ-5D, e.g., as an outcome measure to examine the effect of interventions and used to calculate health utility for economic evaluation. In such case, we categorized the use as cost per QALY.

## Conclusion

This study demonstrates that EQ-5D has been used extensively for a wide variety of purposes, both non-economic and economic. Detailed research with respect to applications in, e.g., specific disease areas, could provide further insight into how EQ-5D has been utilized.
